# Synthesis, Structural Characterization, Luminescent Properties, and Antibacterial and Anticancer Activities of Rare Earth-Caffeic Acid Complexes

**DOI:** 10.3390/molecules30102162

**Published:** 2025-05-14

**Authors:** Nguyen Thi Hien Lan, Hoang Phu Hiep, Tran Van Quy, Pham Van Khang

**Affiliations:** 1 Faculty of Chemistry, Thai Nguyen University of Education, Thai Nguyen City 250000, Vietnam; lannth.chem@tnue.edu.vn (N.T.H.L.); trangiaquy1996@gmail.com (T.V.Q.); 2 Faculty of Biology, Thai Nguyen University of Education, Thai Nguyen City 250000, Vietnam; hiephoangphu@tnue.edu.vn

**Keywords:** rare earth metal, caffeic acid, antibacterial activity, Vietnam, anti-cancer activity

## Abstract

Rare earth elements (Ln: Sm, Eu, Tb, Dy) were complexed with caffeic acid (Caf), a natural phenolic compound, to synthesize novel luminescent complexes with enhanced biological activities. The complexes, formulated as Ln(Caf)_3_·4H_2_O, were characterized using infrared spectroscopy (IR), thermogravimetric analysis (TGA/DTA), mass spectrometry (MS), and fluorescence spectroscopy. Structural studies confirmed the coordination of caffeic acid via carboxylate and hydroxyl groups, forming stable hexacoordinate complexes. Luminescence analysis revealed intense emission bands in the visible spectrum (480–700 nm), attributed to f-f transitions of Ln^3+^ ions, with decay lifetimes ranging from 0.054 to 0.064 ms. Biological assays demonstrated significant antibacterial activity against *Escherichia coli*, *Staphylococcus aureus*, and *Pseudomonas aeruginosa*, with inhibition zones up to 44 mm at 200 µg/mL. The complexes also exhibited potent anticancer activity against MCF7 breast cancer cells, with Sm(Caf)_3_·4H_3_O showing the lowest IC_50_ value (15.5 µM). This study highlights the dual functionality of rare earth metal-caffeic acid complexes as promising candidates for biomedical imaging and therapeutic applications.

## 1. Introduction

Rare earth metals (REMs), particularly the lanthanide series elements, have emerged as a focus of intense research owing to their exceptional physicochemical characteristics, including sharp f-f electronic transitions, high magnetic moments, large coordination numbers, and significant Lewis acidity [1,2]. These properties render them highly attractive for diverse applications in materials science, photonics, sensing, catalysis, and biomedical research [3,4,5]. A key feature of REMs is their strong affinity for oxygen-donor ligands, such as carboxylic acids, phenols, and β-diketones, enabling the formation of thermodynamically stable and structurally versatile coordination complexes [6,7,8]. Among the organic ligands explored, carboxylic acid derivatives, including naturally occurring phenolic acids such as caffeic acid have shown particular promise. These ligands offer multiple donor sites (–COOH, –OH) capable of chelating metal centers, often resulting in the formation of polynuclear or polymeric architectures [9,10,11]. The coordination process can significantly alter the physicochemical and biological behavior of both the metal ion and the ligand, leading to new or enhanced functionalities. In biomedical applications, such synergy is of particular interest, as complexation may enhance the solubility, stability, and membrane permeability of bioactive ligands, while also modulating redox properties and facilitating controlled release of metal ions [12,13]. Furthermore, recent studies have suggested that the combination of REMs with bioactive organic molecules can lead to synergistic or additive effects in antimicrobial, antioxidant, anticancer, and enzyme inhibition assays [4,9,14]. This enhancement is often attributed to increased lipophilicity upon complexation, improved cellular uptake, and potential interactions with biomolecular targets such as DNA, proteins, or microbial membranes. Thus, the design of REM-based complexes with biologically active ligands not only provides a route to multifunctional materials, but also offers significant potential in the development of novel therapeutic agents.

Caffeic acid (3,4-dihydroxycinnamic acid), a naturally occurring hydroxycinnamic acid, has attracted considerable attention due to its strong chelating ability, redox properties, and a wide range of biological activities. In vitro and in vivo evidence has confirmed the wide-ranging physiological and pharmacological activities of caffeic acid and its derivatives [15,16,17,18,19,20,21,22,23,24,25,26]. These bioactivities of caffeic acid exhibited as antibacterial [15], antiviral [16,17], antioxidant [18,19,20,21], anti-inflammatory [15,19], anti-atherosclerotic [15], immunostimulatory [21], antidiabetic [22,26], and cardioprotective effects [23], as well as notable antiproliferative [24], hepatoprotective [23], and anticancer properties [25]. Among these, the anticancer potential, particularly the anti-hepatocellular carcinoma (HCC) activity stands out as especially significant. Caffeic acid also exhibited the suppression of the growth of hepatoma cells by inducing apoptosis, inhibiting cell proliferation, and modulating oxidative stress and inflammatory pathways [16,25]. In addition, caffeic acid serves as a strategic scaffold for the synthesis of amides, esters, and N-containing hybrid compounds, designed to optimize its pharmacological properties and broaden its therapeutic potential [24,25,26,27]. Particularly, metal-caffeic acid complexes are considered promising candidates for enhancing its therapeutic potential and expanding its applicability in biomedical and material sciences [28,29,30,31,32,33,34,35,36,37,38,39]. Iron(III)-caffeic acid complex has been extensively studied for their structural, kinetic, and redox behavior. Kinetic and spectroscopic studies have demonstrated the formation of thermodynamically stable Fe(III)-caffeic acid complex, exhibiting promising antioxidant and iron-chelating properties [28]. Metal-phenolic networks (MPNs) incorporating Fe(III) and caffeic acid have emerged as versatile functional materials, enabling tunable optical and degradation properties, with potential uses in stimuli-responsive coatings and drug delivery [29]. Zinc(II) complex with caffeic acid has also been synthesized and characterized, showing improved antioxidant and antidiabetic activities compared to free caffeic acid. The Zn(II)-caffeic acid complex demonstrated enhanced inhibition of α-amylase and α-glucosidase, supported by in silico docking studies, suggesting its promise as a therapeutic candidate in oxidative stress-related diseases [30]. Furthermore, caffeic acid has been successfully intercalated into layered double hydroxide (LDH) matrices, such as ZnAl–LDH, forming hybrid materials with potential application in cosmetic formulations. These systems showed sustained release, photostability, and biocompatibility, making them attractive for skincare products [31]. Copper(II)-caffeic acid interactions have been explored under physiological conditions to assess their role in modulating oxidative stress. Complex formation with Cu(II) altered the pro-oxidant/antioxidant balance of caffeic acid, providing insights into its dual redox behavior and implications for dietary polyphenol-metal interactions [32]. Recently, Lanthanide complexes, particularly Eu(III)-caffeic acid complex, have gained attention for their antimicrobial activity against both Gram-positive and Gram-negative strains, attributed to increased lipophilicity and cellular uptake following metal chelation [33]. The Eu(III)-caffeinate complex demonstrated significantly enhanced antibacterial activity against both *Escherichia coli* (Gram-negative) and *Bacillus subtilis* (Gram-positive) compared to free caffeic acid and EuCl_3_ alone. The findings of Cota et al. [33] demostrated that coordination with Ln^3+^ ions increases the lipophilicity of the ligand, thereby improving its ability to cross lipid membranes and disrupt cellular processes, ultimately leading to bacterial cell death. According to Tweedy’s chelation theory and Overtone’s concept, increased lipophilicity facilitates the passage of the complex through microbial membranes, which are permeable predominantly to lipophilic substances [34,35,36]. This lipophilicity enhancement likely results from partial sharing of the metal ion’s positive charge with donor atoms, enabling π-electron delocalization across the chelate ring [37]. Additionally, metal–ligand interactions may further alter membrane or cell wall properties, contributing to the increased antimicrobial efficacy compared to the free ligand [34,38,39]. Interestingly, both caffeic acid and its Eu(III) complex exhibited lower activity against *Candida albicans* relative to their effects on bacterial strains. This observation is consistent with previous reports on other Ln^3+^ complexes and is attributed to the structural complexity of the fungal cell wall, which limits compound permeability [34]. Moreover, in the case of alkali metal caffeinates such as potassium caffeinate, a slight preference for activity against Gram-positive bacteria was observed, while activity against Gram-negative bacteria and fungi was considerably lower [34]. Despite these advancements, there are still gaps in the literature regarding the systematic study of REM complexes with caffeic acid, particularly their antimicrobial and anticancer properties. Moreover, the thermal stability and structural characterization of these complexes remain underexplored. This study aims to address these gaps by synthesizing and characterizing Sm, Eu, Tb, and Dy complexes with caffeic acid, and evaluating their antimicrobial and anticancer activities.

## 2. Results and Discussion

### 2.1. Analysis the Content of Rare Earth Ions Ln^3+^ in the Complexes

The complexometric titration method using Arsenazo III indicator was employed to determine the content of rare earth ions (Ln^3+^) in the synthesized complexes. An accurately weighed sample of 0.04 gram of the studied complex was transferred to a Kjeldahl flask. The sample was moistened with a few drops of concentrated H_2_SO_4_ and heated on an electric stove until SO_2_ gas was released. After cooling, 1–2 mL of H_2_O_2_ was added, and the solution was heated again to remove any remaining SO_2_. This process was repeated until a clear solution with the characteristic color of rare earth ions was obtained. The resulting solution was transferred to a 50 mL volumetric flask, diluted to the mark with distilled water, and mixed thoroughly. The complexometric titration was then conducted. At the equivalence point, the solution color changed from deep blue to grape red. The results of content of Ln^3+^ in the complexes were presented in the Table 1.

The data in Table 1 demonstrated that the percentage of Ln^3+^ ions calculated based on the proposed empirical formulas aligns closely with the experimental results.

### 2.2. Analysis the FT-IR Spectroscopy of Complexes

**Figure 1:**
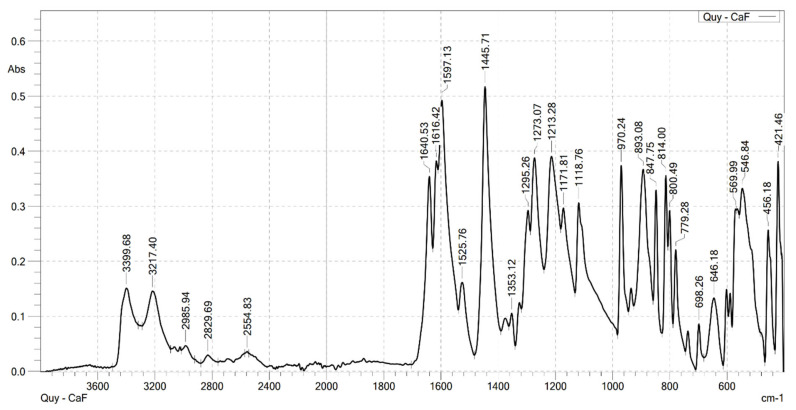
FT-IR spectrum of caffeic acid.

**Figure 2:**
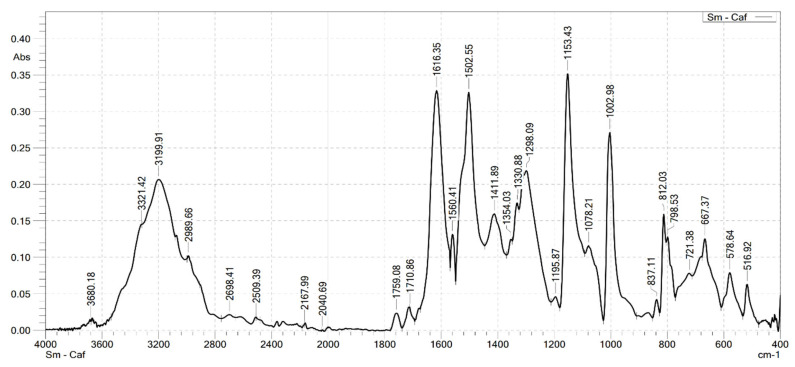
FT-IR spectrum of Sm(Caf)3·4H_2_O.

**Figure 3:**
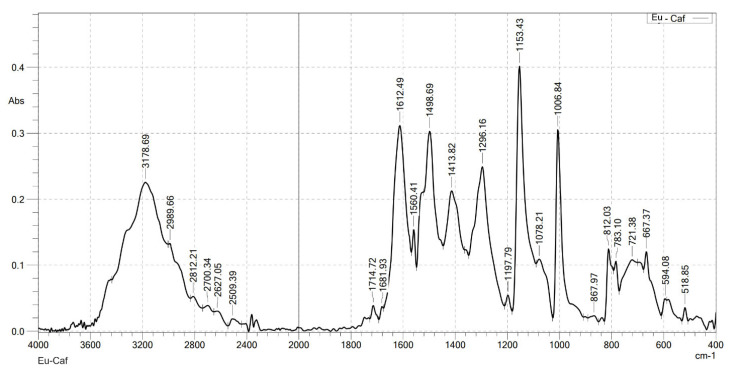
FT-IR spectrum of Eu(Caf)_3_·4H_2_O.

**Figure 4:**
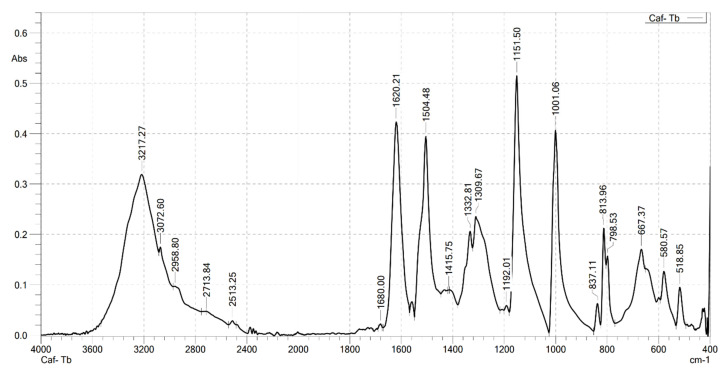
FT-IR spectrum of Tb(Caf)_3_·4H_2_O.

**Figure 5:**
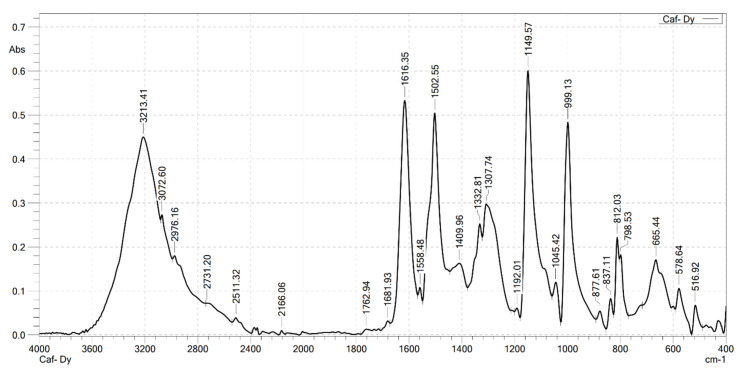
FT-IR spectrum of Dy(Caf)_3_·4H_2_O.

Infrared spectroscopy was used to confirm the formation of the complexes. FT-IR spectra were recorded on a Shimadzu FTIR Affinity-IS spectrometer (Shimadzu, Kyoto, Japan), in the wavenumber range of 400–4000 cm^−1^. The FT-IR spectra of the ligand (caffeic acid) and the four complexes are shown in Figure 1, Figure 2, Figure 3, Figure 4 and Figure 5. Characteristic wavenumbers corresponding to the vibrational modes of functional groups in the compounds are presented in Table 2.

The FT-IR spectrum of free caffeic acid displays a prominent absorption band at 1640 cm^−1^, corresponding to the stretching vibration of the carbonyl (in the carboxylate, –COOH) group, which appears with strong intensity. A broad absorption feature in the range of 3217–3399 cm^−1^ is attributed to the O–H stretching vibrations, indicative of the phenolic hydroxyl and carboxylic acid functionalities [13]. Upon complexation with lanthanide ions (Ln^3+^), significant spectral changes are observed in the FT-IR spectra of the resulting Ln(Caf)_3_·4H_2_O complexes, confirming the formation of coordination bonds between the lanthanide centers and the deprotonated carboxylate ligands. Notably, strong absorption bands emerge in the range of 1612–1620 cm^−1^, which are assigned to the asymmetric stretching vibrations of the carboxylate (–COO^−1^) group. These bands exhibit a marked shift to lower wavenumbers compared to the free ligand (1640 cm^−1^), suggesting coordination of the carboxylate group to the lanthanide ion, which results in electron delocalization and weakening of the C=O bond. In addition, bands observed in the region of 1498–1502 cm^−1^ correspond to the symmetric stretching vibrations of the –COO^−^ group. The presence of both asymmetric and symmetric carboxylate stretching bands, and the difference between them (Δν), further supports the involvement of the carboxylate group in coordination. Vibrational bands appearing in the 578–594 cm^−1^ region are characteristic of metal–oxygen (Ln^3+^–O) stretching modes, providing direct evidence of coordination between the Ln^3+^ center and oxygen donor atoms of the ligand [10]. Furthermore, absorption bands in the range of 2812–2989 cm^−1^ are attributed to the stretching vibrations of aliphatic C–H bonds within the aromatic framework of the lignan moiety [10]. All synthesized complexes also exhibit broad absorption bands in the region of 3199–3217 cm^−1^, which are assigned to O–H stretching vibrations of water molecules. These features indicate the presence of either coordinated or crystallization water molecules within the structures of the complexes. Thus, the FT-IR spectroscopic data confirm that the caffeate ligand coordinates to the Ln^3+^ ion predominantly through its carboxylate moiety, and that all four complexes adopt a similar coordination environment.

### 2.3. Properties of the Thermal Analysis of the Complexes

**Figure 6:**
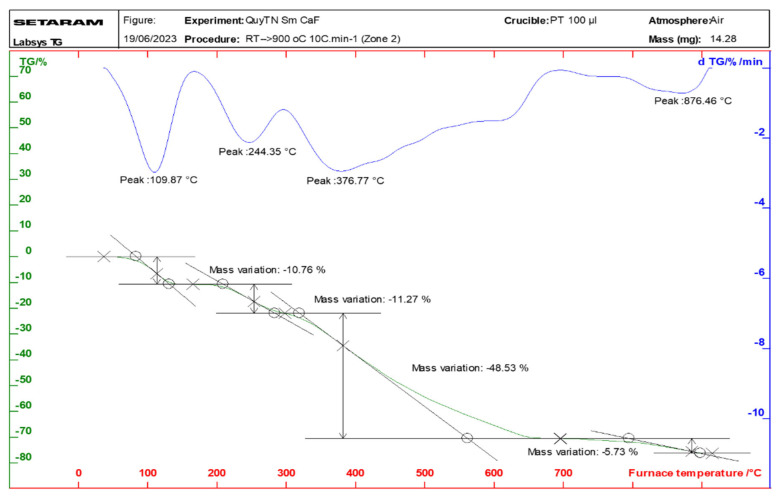
Thermal analysis diagram of Sm(Caf)_3_·4H_2_O complex.

**Figure 7:**
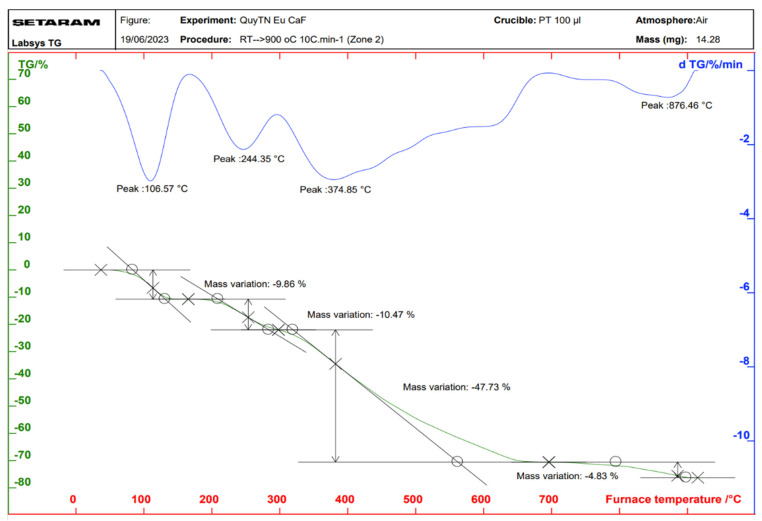
Thermal analysis diagram of Eu(Caf)_3_·4H_2_O complex.

**Figure 8:**
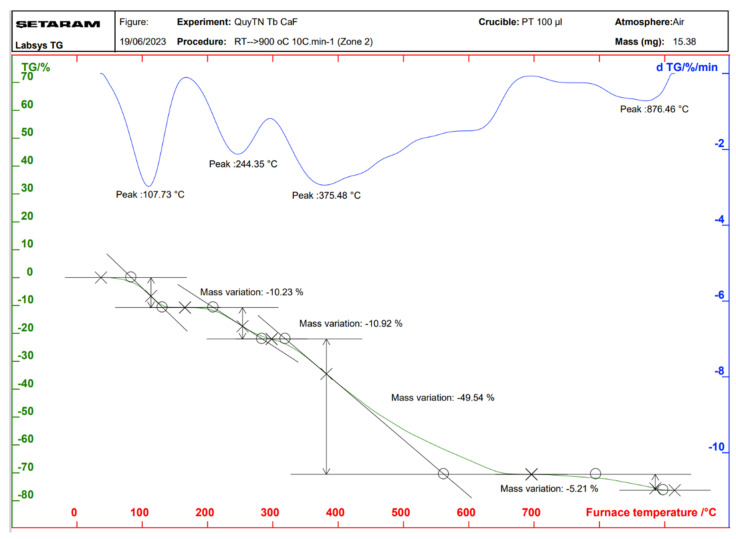
Thermal analysis diagram of Tb(Caf)_3_·4H_2_O complex.

**Figure 9:**
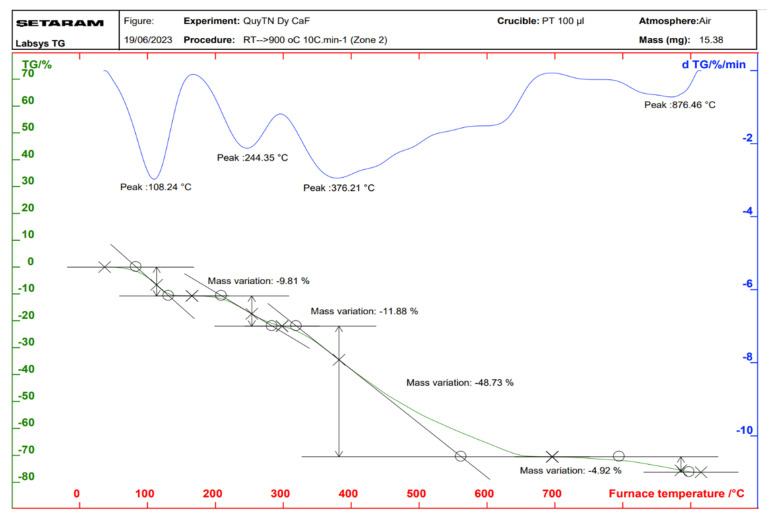
Thermal analysis diagram of Dy(Caf)_3_·4H_2_O complex.

Thermal analysis diagrams of the complexes were recorded using a Themys thermal analyzers (SETARAM KEP Technologies, Caluire, France) in an air atmosphere. The temperature was increased from room temperature to 900 °C at a heating rate of 10 °C/min. Figure 6, Figure 7, Figure 8 and Figure 9 present the thermal analysis curves of the complexes. The results of the thermal decomposition processes are summarized in Table 3.

In the thermal analysis diagrams of the four complexes, a mass loss effect is observed in the temperature range of 106–109 °C, with a weight loss ranging from 9.81% to 10.76%. This is attributed to the removal of four coordinated water molecules (hydration water). These results are in good agreement with the IR spectral data of the complexes. On the TGA curves of the complexes, a significant weight loss is observed starting at approximately 244 °C, corresponding to the initial decomposition of the organic ligand. This decomposition continues until about 876 °C, at which point, the complexes are completely decomposed, resulting in a total weight loss of 63.03–65.67%. The final stable decomposition products are the respective rare earth oxides (Ln_2_O_3_); however, during the thermal decomposition of the Tb(Caf)_3_·4H_2_O complex, an oxidation state as +4 of Tb is formed, leading to the possible generation of Tb_4_O_7_ oxide, and has been demonstrated in the study conducted by Gao et al. [40] and Esra et al. [41]. Theoretical calculations show relatively good agreement with the experimental data obtained.

### 2.4. Mass Spectrum Analysis of the Complexes

In the mass spectra of the four complexes, prominent peaks with the highest *m/z* values were observed at 687, 689, 696, and 699, corresponding to the Sm(III), Eu(III), Tb(III), and Dy(III)-caffeic acid complexes, respectively (Table 4). These values match the molecular ion masses of the rare earth metal-caffeic acid complexes with the formula [Ln(Caf)_3_]^+^ (Ln = Sm, Eu, Tb, Dy). This indicates that under mass spectrometry conditions, after the loss of four hydration water molecules, the resulting molecular ions of the complexes have a consistent structure: [Ln(Caf)_3_]^+^. These data support the proposed molecular formula of the complexes as Ln(Caf)_3_·4H_2_O (Ln = Sm, Eu, Tb, Dy). Mass spectral analysis of the four complexes further revealed that the molecular ion peaks [Ln(Caf)_3_]^+^ are the base peaks, indicating high stability of these molecular ions under the experimental conditions. Additionally, secondary, but still intense peaks were observed at *m*/*z* 508, 510, 517, and 520 for the Sm(Caf)_3_·4H_2_O, Eu(Caf)_3_·4H_2_O, Tb(Caf)_3_·4H_2_O, and Dy(Caf)_3_·4H_2_O complexes, respectively. These peaks were assigned to the presence of fragment monomer ions: [Sm(Caf)_2_]^+^, [Eu(Caf)_2_]^+^, [Tb(Caf)_2_]^+^, and [Dy(Caf)_2_]^+^.

**Figure 10:**
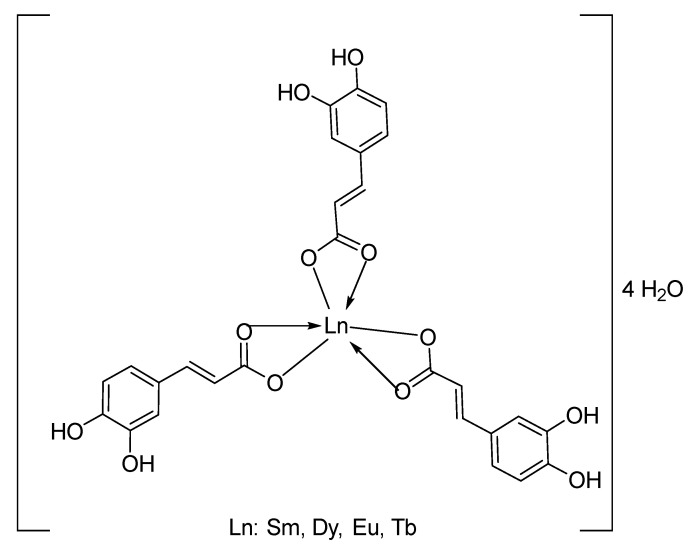
Suggested structural formula for the complexes.

The positive ion mass spectrometry results indicate that the vapor-phase composition of all four complexes is similar, each consisting of a stable monomeric molecular ion [Ln(Caf)_3_]^+^ and a fragment ion [Ln(Caf)_2_]^+^ (Ln = Sm, Eu, Tb, Dy), both of which exhibit significant stability under MS conditions. Based on the mass spectrometry data, IR spectra, and thermal analysis, we propose the following structural formula for the complexes (Figure 10):

### 2.5. Study of Fluorescence Character of Complexes

**Figure 11:**
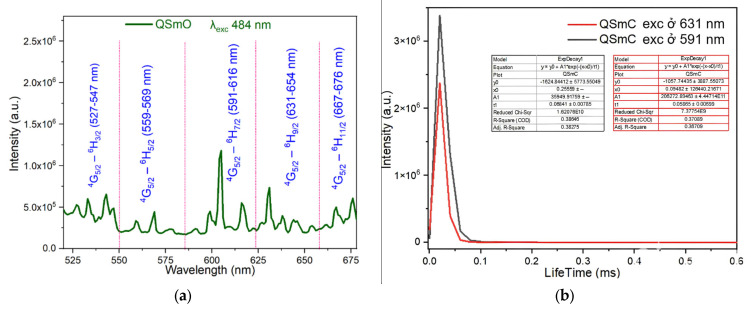
Fluorescence spectrum of the complex Sm(Caf)_3_.4H_2_O (**a**) Fluorescence emission spectrum (**b**) Fluorescence decay spectrum.

**Figure 12:**
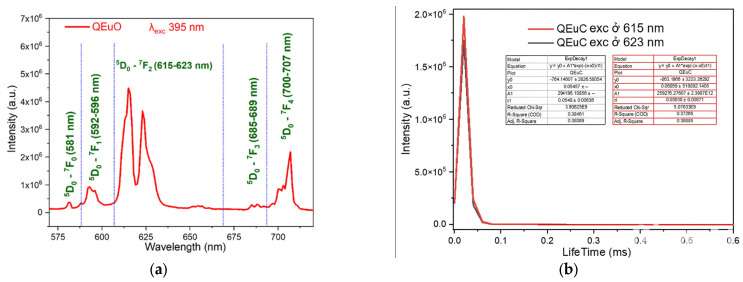
Fluorescence spectrum of the complex Eu(Caf)_3_·4H_2_O. (**a**) Fluorescence emission spectrum; (**b**) Fluorescence decay spectrum.

**Figure 13:**
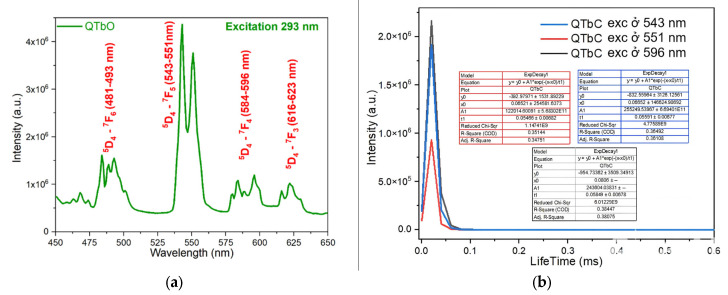
Fluorescence spectrum of the complex Tb(Caf)_3_·4H_2_O. (**a**) Fluorescence emission spectrum (**b**) Fluorescence decay spectrum.

**Figure 14:**
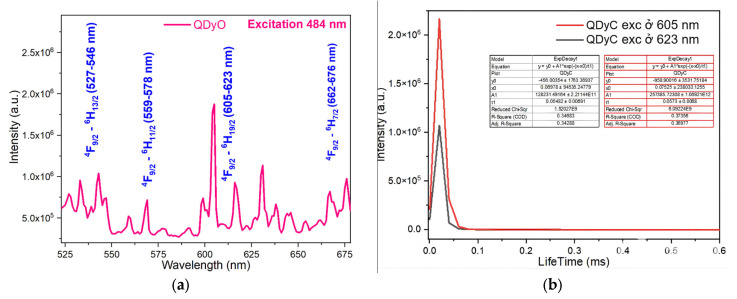
Fluorescence spectrum of the complex Dy(Caf)_3_·4H_2_O. (**a**) Fluorescence emission spectrum (**b**) Fluorescence decay spectrum.

The fluorescence emission spectra of the synthesized complexes were recorded using a spectrophotometer. The fluorescence emission spectra and fluorescence decay spectra of the complexes are presented in Figure 11, Figure 12, Figure 13 and Figure 14.

Upon excitation at wavelength 484 nm, the fluorescence emission spectrum of the Sm(Caf)_3_·4H_2_O complex displays five distinct emission bands in the visible region, specifically within the ranges of 527–547 nm (green), 559–569 nm (green), 591–616 nm (orange-yellow), 631–654 nm (red), and 667–676 nm (deep red). These emission bands are assigned to the characteristic 4f-4f electronic transitions of the Sm^3+^ ion, corresponding to the transitions ^4^G_5_/_2_ → ^6^H_3_/_2_, ^4^G_5_/_2_ → ^6^H_5_/_2_, ^4^G_5_/_2_ → ^6^H_7_/_2_, ^4^G_5_/_2_ → ^6^H_9_/_2_, and ^4^G_5_/_2_ → ^6^H_11_/_2_, respectively [42,43,44,45]. Among these, the ^4^G_5_/_2_ → ^6^H_7_/_2_ transition, located in the orange-yellow region (591–616 nm), exhibits the highest emission intensity, consistent with the hypersensitive nature of this transition and its strong dependence on the coordination environment of the Sm^3+^ ion. To further investigate the photophysical behavior of the complex, time-resolved fluorescence measurements were conducted. The fluorescence decay profiles were recorded under excitation at 484 nm, with emission monitoring at 591 nm and 631 nm, corresponding to the ^4^G_5_/_2_ → ^6^H_7_/_2_ and ^4^G_5_/_2_ → ^6^H_9_/_2_ transitions, respectively. The decay curves were fitted using a mono-exponential model, yielding fluorescence lifetimes of 0.0641 ± 0.00785 ms and 0.05865 ± 0.00699 ms, respectively (Figure 11b). These lifetime values reflect efficient intramolecular energy transfer processes and confirm the emissive nature of the Sm^3+^ center within the caffeinate ligand field [44,45].

For Eu(Caf)_3_·4H_2_O complex, under excitation at wavelength 395 nm (Figure 12a), it exhibits five characteristic emission bands corresponding to Eu^3+^ ion transitions. These emission bands are assigned to the following transitions: ^5^D_0_ → ^7^F_0_ (581 nm), ^5^D_0_ → ^7^F_1_ (592–596 nm), ^5^D_0_ → ^7^F_2_ (615–623 nm), ^5^D_0_ → ^7^F_3_ (685–689 nm), and ^5^D_0_ → ^7^F_4_ (700–707 nm) [42,43,44,45]. These transitions correspond to emissions in the green region (581 nm), orange-yellow region (592–596 nm and 615–623 nm), and red region (685–689 nm and 700–707 nm). Among these, the orange-yellow emission band attributed to the ^5^D_0_ → ^7^F_2_ transition of the Eu^3+^ ion exhibits the strongest intensity [23]. The luminescence decay of the Eu(Caf)_3_·4H_2_O complex was monitored at emission wavelengths of 615 nm and 623 nm. The corresponding fluorescence lifetimes were determined to be 0.05638 ± 0.00671 ms and 0.05480 ± 0.00638 ms, respectively (Figure 12b). These two emission bands originate from the radiative transition of the excited Eu^3+^ ion, specifically from the ^5^D_0_ → ^7^F_J_ manifold. The emission at 615 nm corresponds to the hypersensitive ^5^D_0_ → ^7^F_2_ transition, which is highly influenced by the local ligand field asymmetry. The emission at 623 nm, while less intense, may arise from crystal field splitting of the ^7^F_2_ level or involve a minor contribution from the ^5^D_0_ → ^7^F_3_ transition, depending on the coordination environment. The observed lifetimes are relatively close in value, indicating that both emissions likely originate from the same excited electronic state (^5^D_0_) of Eu^3+^. The small difference in decay times (~1.58 μs) may reflect subtle variations in radiative and non-radiative deactivation pathways, possibly influenced by the electronic properties of the caffeine ligands and the geometry of the coordination sphere. These results suggest that the Eu(Caf)_3_·4H_2_O complex exhibits typical luminescent behavior of Eu^3+^-based coordination compounds, with relatively long-lived excited states in the microsecond range, suitable for time-resolved luminescence applications.

The photophysical behavior of the terbium(III) complex, Tb(Caf)_3_·4H_2_O, was investigated under ultraviolet excitation at 293 nm. The complex displays characteristic emissions of the Tb^3+^ ion, corresponding to the intra-4f electronic transitions from the excited state ^5^D_4_ to the lower-lying ^7^F_J_ levels. Specifically, four emission bands were observed at the following wavelength ranges: ^5^D_4_ → ^7^F_6_ (481–493 nm), ^5^D_4_ → ^7^F_5_ (543–551 nm), ^5^D_4_ → ^7^F_4_ (584–596 nm), and ^5^D_4_ → ^7^F_3_ (616–623 nm) [43,44,45]. These emissions fall within the visible region, producing violet-blue, green, and red luminescence, respectively. Among these, the ^5^D_4_ → ^7^F_5_ transition exhibits the highest intensity, confirming its dominance in the radiative deactivation pathway of the Tb^3+^ ion. To further assess the excited-state dynamics, time-resolved luminescence measurements were conducted at selected emission wavelengths (Figure 13a). Under excitation at 293 nm, the fluorescence decay profiles at 543 nm, 551 nm, and 596 nm yielded lifetimes of 0.05591 ± 0.00677 ms, 0.05466 ± 0.00682 ms, and 0.05849 ± 0.00678 ms, respectively (Figure 13b). The similarity in decay times, all within the range of ~0.055 ms, indicates that the emissions originate from a common excited state (^5^D_4_) and are minimally influenced by non-radiative quenching under the experimental conditions. These findings confirm the efficient sensitization of Tb^3+^ luminescence by the caffeic acid ligands and highlight the potential of Tb(Caf)_3_·4H_2_O as a green-emitting phosphorescent material with microsecond-scale emission lifetimes, suitable for use in optoelectronic devices and luminescent probes.

To further elucidate the excited-state dynamics of the Dy^3+^ ion in the Dy(Caf)_3_·4H_2_O complex, fluorescence decay measurements were performed at selected emission wavelengths corresponding to its principal transitions. Under excitation at 484 nm (Figure 14a), the fluorescence emissions were monitored at 546 nm, 578 nm, and 613 nm, corresponding respectively to the ^4^F_9_/_2_ → ^6^H_13_/_2_, ^4^F_9_/_2_ → ^6^H_11_/_2_, and ^4^F_9_/_2_ → ^6^H_9_/_2_ transitions. The decay profiles were found to follow a mono-exponential behavior, indicating a single dominant emissive species. The extracted lifetimes were in the range of ~0.05–0.06 ms, consistent with parity-forbidden 4f-4f transitions typical of lanthanide ions in low-symmetry environments. Notably, the emission at 613 nm (^4^F_9_/_2_ → ^6^H_9_/_2_), which exhibited the highest steady-state intensity, also showed the longest lifetime, supporting the efficient population of this radiative channel. The relatively long lifetimes and narrow emission bands observed in the Dy^3+^ complex are indicative of a well-protected 4f electronic environment, likely facilitated by the coordination of caffeic acid ligands, which suppress non-radiative quenching by high-energy vibrational modes (e.g., O–H and N–H stretching). These findings highlight the suitability of Dy(Caf)_3_·4H_2_O for use in time-gated luminescent applications, such as bioimaging and lanthanide-based sensing. Additional lifetime measurements were performed at 605 nm and 623 nm, corresponding to the ^4^F_9_/_2_ → ^6^H_9_/_2_ transition of Dy^3+^, under excitation at 484 nm. The observed fluorescence decay times were 0.0573 ± 0.0068 ms and 0.05482 ± 0.00691 ms, respectively (Figure 14b). These results are consistent with the monoexponential decay behavior observed for other Dy^3+^-centered emissions in the complex, and the similarity in lifetime values further confirms that both emissions originate from the same excited electronic state (^4^F_9_/_2_). The minor variation in lifetime between the two wavelengths may reflect subtle differences in radiative probabilities or local symmetry perturbations imposed by the ligand field. Overall, the fluorescence lifetimes of Dy(Caf)_3_·4H_2_O fall within the expected microsecond range for forbidden 4f-4f transitions and demonstrate that the coordination of caffeic acid provides a sufficiently rigid and low-vibrational environment to preserve excited-state integrity. This enhances the radiative efficiency of the Dy^3+^ ion and supports the potential utility of this complex in time-resolved luminescence-based applications.

### 2.6. The Antimicrobial Activity

**Figure 15:**
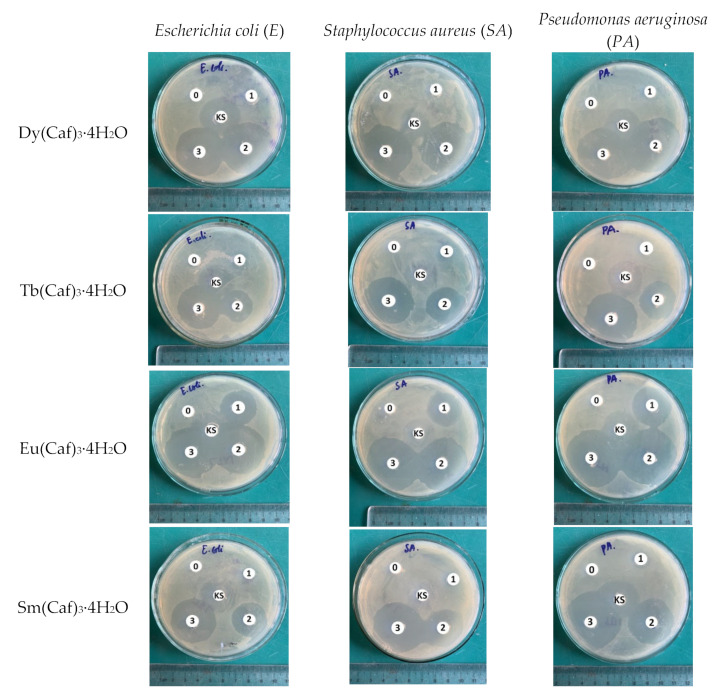
The antimicrobial activity of the synthesized complexes.

The antimicrobial activity of the synthesized complexes was assessed against representative Gram-positive and Gram-negative bacterial strains, including *Escherichia coli* (*E. coli*), *Staphylococcus aureus* (*S. aureus*), and *Pseudomonas aeruginosa* (*P. aeruginosa*). All bacterial strains were obtained from the Department of Biology, Thai Nguyen University of Education, Vietnam National University (TNU). The antimicrobial efficacy of the complexes was evaluated using disc agar diffusion method and the results are summarized in Table 5 and illustrated in Figure 15.

The results clearly indicate that the antibacterial activity of the complexes is both compound-dependent and concentration-dependent. Among the tested complexes, Eu(Caf)_3_·4H_2_O exhibited the strongest inhibitory effect across all bacterial strains, with inhibition zones reaching 40 mm (*E. coli*), 44 mm (*S. aureus*), and 42 mm (*P. aeruginosa*) at 200 µg/mL, surpassing the standard antibiotic amoxicillin in several cases. Dy(Caf)_3_·4H_2_O showed comparable activity at higher concentrations, with inhibition zones of 38 mm, 40 mm, and 38 mm against *E. coli*, *S. aureus*, and *P. aeruginosa*, respectively, at 200 µg/mL. Tb(Caf)_3_·4H_2_O displayed moderate antibacterial activity, reaching maximum inhibition zones of 35–36 mm at the highest concentration. In contrast, Sm(Caf)_3_·4H_2_O demonstrated the weakest antibacterial effect, with inhibition zones not exceeding 31 mm, and no observable activity at 50 µg/mL. At the lowest concentration (50 µg/mL), most complexes, with the exception of Eu, exhibited no measurable inhibition, emphasizing the importance of concentration in achieving biological efficacy. The data suggest that the enhanced antibacterial properties may stem from the ability of lanthanide ions, particularly Eu^3+^ and Dy^3+^, to disrupt bacterial cell membranes, potentially through interaction with mesosomal structures involved in critical functions such as DNA replication and respiration [33]. The antimicrobial activity observed for the synthesized lanthanide-caffeic acid complexes aligns with findings from previous studies investigating similar lanthanide-phenolic acid complexes.

In the present study, the Eu(Caf)_3_·4H_2_O complex exhibited significant antibacterial activity, with inhibition zones reaching up to 44 mm against *Staphylococcus aureus* at a concentration of 200 µg/mL. This potency surpasses that of standard antibiotics like amoxicillin, which showed inhibition zones of 23 mm under similar conditions. Such findings suggest that the Eu(III)-caffeic acid complex possesses superior antibacterial efficacy, potentially due to its unique structural and electronic properties. Furthermore, the observed trend in antibacterial activity among the synthesized complexes-Eu > Dy > Tb > Sm correlates with the findings of Arciszewska et al. [33], where Eu(III) and Dy(III) complexes displayed more pronounced antimicrobial effects compared to other lanthanide counterparts. This consistency across studies underscores the potential of specific lanthanide ions, particularly Eu(III) and Dy(III), in developing effective antimicrobial agents. The enhanced activity of these complexes may be attributed to the ability of lanthanide ions to disrupt bacterial cell membranes, interfere with mesosomal structures involved in vital cellular processes, and increase the permeability of the bacterial cell wall. These mechanisms collectively contribute to the observed antibacterial efficacy and warrant further investigation into the therapeutic potential of lanthanide-ligand complexes.

### 2.7. Anticancer Activity

The cytotoxic activity of the lanthanide-caffeic acid complexes Ln(Caf)_3_·4H_2_O (Ln = Eu, Dy, Tb, Sm) was evaluated against the human breast cancer cell line MCF7 using the MTT assay. Cells were seeded in 96-well plates at a density of 5 × 10^3^ cells/cm^2^ and treated with various concentrations of the complexes (0, 0.05, 0.1, and 0.2 mg/mL) for 48 h. Cell viability was determined spectrophotometrically and expressed as a percentage relative to the untreated control. The half-maximal inhibitory concentration (IC_50_) values were calculated using GraphPad Prism 5.0, and the results are summarized in Table 6. The data revealed that all four lanthanide-caffeic acid complexes exhibited significant inhibitory effects on MCF7 cell proliferation, with IC_50_ values ranging from 15.5 µM to 23.2 µM. Among the tested complexes, Sm(Caf)_3_·4H_2_O showed the highest anticancer potency, with an IC_50_ of 15.5 µM, followed closely by Tb(Caf)_3_·4H_2_O at 16.5 µM and Eu(Caf)_3_·4H_2_O at 20.4 µM. Dy(Caf)_3_·4H_2_O demonstrated slightly lower cytotoxicity, with an IC_50_ of 23.2 µM. When compared to the free ligand caffeic acid (IC_50_ = 27.2 µM), all metal complexes exhibited enhanced anticancer activity, confirming that coordination with lanthanide ions significantly improved the cytotoxic potential of the parent ligand. However, the cytotoxic effects of these complexes were still moderate compared to the standard anticancer agent ellipticine (IC_50_ = 0.23 µM), used as a positive control. The improved activity of the lanthanide-caffeic acid complexes can be attributed to the increased lipophilicity and cellular uptake of the metal-igand complex, as well as the ability of lanthanide ions to disrupt intracellular targets, such as DNA or mitochondrial function. The strong inhibitory activity of Sm(III) and Tb(III) complexes suggests these ions may possess favorable coordination geometries or electronic configurations that facilitate interactions with key biomolecular targets in cancer cells.

These results align with previous studies that reported enhanced cytotoxicity of metal–caffeate complexes compared to the free ligand. For example, hexyl caffeate and caffeoylhexylamide, lipophilic derivatives of caffeic acid, showed improved antiproliferative activity against MCF7 cells, attributed to increased cellular permeability and stability [46]. Similarly, studies on caffeic acid and gallic acid revealed that although both compounds exhibit anticancer activity, the IC_50_ of caffeic acid was approximately 880 µM [47], much higher than the IC_50_ values observed in this study for the Ln(Caf)_3_·4H_2_O complexes. Moreover, the cytotoxic mechanism of phenolic acids such as caffeic acid often involves modulation of key apoptotic markers including P53, Mcl-1, and P21 [46], suggesting that Ln-based complexes may potentiate similar or enhanced pathways due to metal-mediated intracellular signaling or membrane interactions. These findings support the hypothesis that coordination of caffeic acid with lanthanide ions not only retains, but amplifies its bioactivity, potentially offering a dual mode of action-metal ion interaction and phenolic-induced apoptosis. The stronger activity of the Sm(III) complex may also reflect unique electronic configurations or binding affinities of Sm^3+^ with cellular biomolecules. Samarium (Sm^3+^) has an electronic configuration of [Xe]4f^5^, while europium (Eu^3+^) is [Xe]4f^6^, terbium (Tb^3+^) is [Xe]4f⁸, and dysprosium (Dy^3+^) is [Xe]4f⁹. The lower number of f electrons in Sm^3+^ leads to reduced shielding and more accessible coordination sites compared to the heavier-loaded f orbitals of Eu^3+^, Tb^3+^, and Dy^3+^ ions. Consequently, Sm^3+^ can form more stable and compact coordination complexes with caffeic acid, resulting in stronger metal-ligand bonding. This stronger binding enhances the overall structural stability of the complex and likely improves its bioavailability and biological interaction, thus leading to the observed enhancement in bioactivity. Additionally, Sm^3+^’s relatively larger ionic radius compared to Tb^3+^ and Dy^3+^ (but comparable to Eu^3+^) also plays a role in optimizing the coordination environment, balancing flexibility and rigidity, and further contributing to its superior efficiency [48,49,50,51]. Further mechanistic studies, including cellular uptake analysis, ROS measurements, and protein expression profiling, are warranted to fully elucidate the anticancer pathways involved [46]. Nonetheless, these lanthanide-caffeic acid complexes represent promising scaffolds for the design of new metal-based anticancer therapeutics.

## 3. Materials and Methods

### 3.1. Synthesis of Ln(Caf)3·4H_2_O Complexes

To prepare LnCl_3_ solutions, 2.0 × 10^−4^ mol of Ln_2_O_3_ oxides were dissolved in 10 mL of concentrated hydrochloric acid (36.5%). The mixture was stirred and heated at a temperature of approximately 65–70 °C. After about 40 min, a clear solution containing LnCl_3_ and excess HCl was obtained. The excess acid was removed, and the mixture was heated further until anhydrous LnCl_3_ salt was obtained. This salt was then dissolved in distilled water to yield an aqueous solution of LnCl_3_.

To establish a synthetic procedure for Ln(III)-caffeic acid complexes, we investigated factors influencing the complexation process such as temperature and pH. The optimal conditions for complex synthesis were determined to be a pH of approximately 4–5 and a temperature of 50 °C. The Ln(III) caffeniate complexes were synthesized according to the following procedure: 0.216 g (1.2 × 10^−3^ mol) of caffeic acid was completely dissolved in ethanol. A solution of LnCl_3_ (4.0 × 10^−4^ mol) was then added to the ligand solution at a molar ratio appropriate for complexation. The resulting mixture was stirred using a magnetic stirrer with heating at 50 °C and maintained at a pH of approximately 4–5. The time required for precipitate formation varied depending on the specific lanthanide ion. For Sm(III) and Eu(III) complexes, the precipitate appeared after approximately 2.5 h. For the Tb(III) complex, the precipitate formed after approximately 4 h. For the Dy(III) complex, the precipitate appeared after approximately 4.5 h. Upon appearance of the precipitate, stirring was continued at room temperature until no further increase in precipitate amount was observed. The resulting solid was filtered and washed with distilled water using a vacuum filtration equipment. The complexes were then dried in a desiccator until constant mass was achieved.

### 3.2. Antibacterial Activity

The antibacterial properties of the synthesized lanthanide–caffeniate as Ln(Caf)_3_·4H_2_O (Ln = Sm, Eu, Tb, Dy) complexes were assessed against both Gram-negative (*Escherichia coli* and *Pseudomonas aeruginosa*) and Gram-positive (*Staphylococcus aureus*) bacterial strains. All microbial strains were obtained from the Faculty of Biology, Thai Nguyen University of Education. Antibacterial activity was evaluated using the standard agar well diffusion method. LB nutrient medium was prepared with the following composition: tryptone (10 g), NaCl (10 g), yeast extract (5 g), and distilled water (950 mL). The bacterial strains were cultured at 30 °C for 24 h, and microbial suspensions were adjusted to a concentration of approximately 10^8^ CFU/mL using sterile distilled water. A total volume of 0.1 mL of each microbial suspension was uniformly spread over the surface of LB agar plates. Wells of 6 mm in diameter were punched into the agar using a sterile cork borer. Each well was loaded with 50 µL of the test complex solution at three concentrations: 50 µg/mL, 100 µg/mL, and 200 µg/mL, prepared in DMSO. Plates were pre-incubated at 4 °C for 1 h to allow diffusion, then incubated at 37 °C for 24 h. The antibacterial activity was determined by measuring the diameter (mm) of the inhibition zones formed around the wells. Amoxycillin at 50 µg/mL served as the positive control, while DMSO alone was used as the negative control. Each experiment was conducted in triplicate, and mean inhibition zone diameters were reported with standard deviations [52].

### 3.3. Cytotoxicity Assay

The cytotoxic effects of the lanthanide-caffeniate as Ln(Caf)_3_·4H_2_O (Ln = Sm, Eu, Tb, Dy) complexes were examined against the MCF7 (human breast cancer cell) line from Sigmaaldrich, Singapore (Code 41106514) using the MTT (3-(4,5-dimethylthiazol-2-yl)-2,5-diphenyltetrazolium bromide) assay [53]. MCF7 cells were cultured in Dulbecco’s Modified Eagle Medium (DMEM) supplemented with 10% fetal bovine serum (FBS), L-glutamine, sodium pyruvate, NaHCO_3_, and 1% penicillin–streptomycin. Cells were seeded into 96-well plates at a density of 5 × 10^3^ cells/cm^2^ and incubated for 24 h at 37 °C in a humidified atmosphere containing 5% CO_2_. The cells were then treated with varying concentrations of the test complexes for 48 h. After treatment, 20 µL of MTT solution (5 mg/mL in PBS) was added to each well and incubated for an additional 4 h. The resulting formazan crystals were dissolved in 100 µL of DMSO, and absorbance was measured at 550 nm using a microplate reader. Cell viability was expressed as a percentage relative to the untreated control group using the formula:

$$Cell\;viability\left(\%\right)=\left(\frac{{OD}_{treated}}{{OD}_{control}}\right)\times 100$$

The half-maximal inhibitory concentration (IC_50_) values were determined using GraphPad Prism 5.0 software with nonlinear regression analysis.

### 3.4. Statistical Analysis

All experiments were performed in triplicate, and data are presented as mean ± standard deviation (SD). Statistical significance was assessed using two-way analysis of variance (ANOVA), and a *p*-value less than 0.05 was considered statistically significant.

## 4. Conclusions

In summary, this study presents a comprehensive synthesis and characterization of novel lanthanide-caffeic acid complexes, Ln(Caf)_3_·4H_2_O (Ln = Sm, Eu, Tb, Dy), with emphasis on their structural, photophysical, and biological properties. Spectroscopic, thermal, and mass spectrometric analyses confirmed the successful coordination of caffeic acid through carboxylate moiety, resulting in stable hexacoordinate complexes. Luminescence studies revealed intense f-f emission transitions with microsecond-range decay lifetimes, supporting their potential as photoluminescent materials for time-resolved imaging. Biologically, the complexes exhibited potent antimicrobial activity, particularly the Eu(III) complex, which outperformed standard antibiotics against multiple bacterial strains, as well as significant cytotoxicity against MCF7 breast cancer cells, with Sm(III) complex showing the lowest IC_50_ value. These findings underscore the dual functionality of rare earth metal-caffeic acid complexes and their promising applications in biomedical diagnostics and therapeutics. Further exploration into their mechanisms of action and in vivo performance is warranted to advance their translational potential.

## Figures and Tables

**Table 1:** Percentage content of Ln^3+^ in the complexes.

Complex	Theoretical Ln^3+^ Content (%)	Experimental Ln^3+^ Content (%)
Sm(Caf)_3_·4H_2_O	19.78	19.75
Eu(Caf)_3_·4H_2_O	19.95	19.94
Tb(Caf)_3_·4H_2_O	20.68	20.64
Dy(Caf)_3_·4H_2_O	21.04	21.05

**Table 2:** Characteristic absorption wavenumbers in the FT-IR spectrum of ligand and complexes (cm^−1^).

Compounds	*v*_(COOH)_	ν_as(COO_–_)_	ν_s(COO_–_)_	*v*_(Ln–O)_	*v*_(OH)_	*v_(_*_CH)_
HCaf	1640	–	1445	–	3217 3399	2985
Sm(Caf)_3_.4H_2_O	–	1616	1502	578	3199	2989
Eu(Caf)_3_.4H_2_O	–	1612	1498	594	3178	2812
Tb(Caf)_3_.4H_2_O	–	1620	1504	580	3217	2958
Dy(Caf)_3_.4H_2_O	–	1616	1502	578	3213	2976

**Table 3:** Results of thermogram analysis of complexes.

Complex	Temperature at Which the Mass Loss Effect Occurs (°C)	Process	The Remaining Structure	Loss Weight (%)	
				Theory	Experiment
Sm(Caf)_3_·4H_2_O	109	Remove water	Sm(Caf)_3_	9.47	10.76
	244	Decomposition	Sm_2_O_3_	67.63	65.53
	376				
	876				
Eu(Caf)_3_·4H_2_O	106	Remove water	Eu(Caf)_3_	9.45	9.86
	244	Decomposition	Eu_2_O_3_	67.43	63.03
	374				
	876				
Tb(Caf)_3_·4H_2_O	107	Remove water	Tb(Caf)_3_	9.36	10.23
	244	Decomposition	Tb_4_O_7_	66.31	65.67
	375				
	876				
Dy(Caf)_3_·4H_2_O	108	Remove water	Dy(Caf)_3_	9.32	9.81
	244	Decomposition	Dy_2_O_3_	66.62	65.53
	376				
	876				

**Table 4:** Mass spectrum data of the complexes.

Complex	Ion Fragment	*m*/*z*	(%)
Sm(Caf)_3_·4H_2_O	[Sm(Caf)_3_]^+^	687	100
	[Sm(Caf)_2_]^+^	508	75.71
Eu(Caf)_3_·4H_2_O	[Eu(Caf)_3_]^+^	689	100
	[Eu(Caf)_2_]^+^	510	73.91
Tb(Caf)_3_·4H_2_O	[Tb(Caf)_3_]^+^	696	100
	[Tb(Caf)_2_]^+^	517	88.40
Dy(Caf)_3_·4H_2_O	[Dy(Caf)_3_]^+^	699	100
	[Dy(Caf)_2_]^+^	520	47.14

**Table 5:** The antimicrobial activity of the synthesized complexes.

Sample	Concentration	*Escherichia coli* (E.)	*Staphylococcus aureus* (SA)	*Pseudomonas aeruginosa* (PA)
Amoxicillin	50 µg/mL	30	23	25
Dy(Caf)_3_·4H_2_O	50 µg/mL	-	-	-
	100 µg/mL	35	37	35
	200 µg/mL	38	40	38
Tb(Caf)_3_·4H_2_O	50 µg/mL	-	-	-
	100 µg/mL	25	28	26
	200 µg/mL	35	36	36
Eu(Caf)_3_·4H_2_O	50 µg/mL	25	29	26
	100 µg/mL	36	43	40
	200 µg/mL	40	44	42
Sm(Caf)_3_·4H_2_O	50 µg/mL	-	-	-
	100 µg/mL	18	18	25
	200 µg/mL	29	30	31

**Table 6:** Inhibitory Effects Of The Complexes On MCF7 Cancer Cell Line Of Ln(Caf)_3_·4H_2_O.

Complex	IC_50_ (µM)
Dy(Caf)_3_·4H_2_O	23.2 ± 0.5
Eu(Caf)_3_·4H_2_O	20.4 ± 0.4
Sm(Caf)_3_·4H_2_O	15.5 ± 0.3
Tb(Caf)_3_·4H_2_O	16.5 ± 0.6
Caffeic acid	27.2 ± 0.7
Elippticine (control)	0.23 ± 0.8

## Data Availability

The original contributions presented in the study are included in the article, further inquiries can be directed to the corresponding author.
